# Pragmatic approaches for addressing alcohol in general practice: Development of a tailored implementation intervention

**DOI:** 10.3389/frhs.2022.940383

**Published:** 2022-11-17

**Authors:** Sebastian Potthoff, Amy Jane O'Donnell, Andrea Taksdal Karlsen, Håvar Brendryen, Torgeir Gilje Lid

**Affiliations:** ^1^Centre for Alcohol and Drug Research, Stavanger University Hospital, Stavanger, Norway; ^2^Department of Social Work, Education and Community Wellbeing, Northumbria University, Newcastle upon Tyne, United Kingdom; ^3^Population and Health Sciences Institute, Newcastle University, Newcastle upon Tyne, United Kingdom; ^4^Department of Psychology, Faculty of Social Sciences, University of Oslo, Oslo, Norway; ^5^Faculty of Health Sciences, University of Stavanger, Stavanger, Norway; ^6^Research Unit for General Practice, NORCE Norwegian Research Centre, Bergen, Norway

**Keywords:** alcohol, targeted screening, brief intervention, implementation, implementation determinants, tailoring, behavior change, behavior change wheel

## Abstract

**Introduction:**

Alcohol consumption is a leading global risk factor for ill-health and premature death. Alcohol screening and brief interventions (SBI) delivered in primary care is effective at reducing alcohol consumption, but routine implementation remains problematic. Screening all patients for excessive drinking (universal screening) is resource-intensive and may be at odds with general practitioners' (GPs') perceived professional role. This study aimed to develop a tailored, theory-based training intervention to strengthen GPs' ability to address alcohol and to manage alcohol-related health problems through a pragmatic approach based on clinical relevance.

**Methods:**

A qualitative study design involving focus group interviews and a structured questionnaire for free text replies with GPs in Norway. Behavioral analysis assessed factors influencing delivery of SBI according to the ‘capability, opportunity, motivation and behavior' (COM-B) model to inform intervention development using the Behavior Change Wheel. Qualitative data were analyzed using framework analysis and an iterative approach was adopted to develop the training.

**Results:**

A purposive sample of GPs attended the focus groups (*n* = 25) and completed the questionnaire (*n* = 55). Four areas required additional support including: understanding the link between alcohol use and health problems; opening up the conversation on alcohol use; addressing alcohol and dealing with obstacles; and following-up and maintaining change. Findings informed the development of a four-session interactive training intervention and a digital intervention for providing support for patients between consultations to address the identified needs.

**Conclusion:**

This work highlights the value of pragmatic, relevance-based clinical strategies, as opposed to universal screening approaches to addressing alcohol in primary care. A pragmatic approach is more in line with GPs existing sclinical skill set and holds the potential to improve widespread uptake and implementation of SBI in routine primary care.

## Introduction

Excessive alcohol consumption is a leading health risk worldwide (1). In 2020, alcohol sales were above seven liters of pure alcohol per person in Norway, which is a 19.5% increase from 2019 and record high (2). Excessive alcohol consumption is associated with a range of adverse health, social and economic consequences (3). Screening and brief intervention (SBI) can help reduce alcohol consumption and has the potential to reach a large proportion of the population, at relatively low cost (4, 5). SBI involves first using a validated questionnaire to identify patients drinking at a hazardous or harmful level (6), followed by the delivery of a brief behavioral intervention to those needing support. Despite proven effectiveness of brief alcohol interventions, both face-to-face and when delivered digitally (4, 7), there has been limited implementation of SBI in primary care systems globally (8, 9).

Multiple barriers exist to widespread implementation of SBI in primary health care, including individual level (practitioner or patient), organizational (practice) and wider health system factors (10, 11). At an individual level, previous evidence suggests that general practitioners (GPs) find it more challenging to deliver SBI to specific patient groups (12), and fear alienating their patients by raising a potentially sensitive topic in routine consultations (13). GPs can lack the necessary knowledge about the connection between alcohol and related health problems, and may have limited access to evidence-based strategies and tools for intervening with patients when relevant (10). For example, a cross-sectional study in Norway found limited knowledge and use of screening tools in Norwegian GPs (14). A subsequent qualitative study identified a range of barriers to implementing such interventions, such as the challenge of integrating SBI into existing clinical routines or being worried about disrupting the doctor-patient relationship (15).

Moreover, GPs are often resistant toward universal, population-based approaches to SBI, presumably because of their laborious and time-consuming nature (16). Targeted or symptom-based approaches are more acceptable to GPs, and evidence suggests that targeted screening may be more efficient as it yields a higher prevalence of at-risk alcohol consumers than universal screening (17). Several strategies based on clinical relevance have been reported, e.g., semi-systematic method, pragmatic case findings, and relevance criteria, the latter focused on smoking but used a similar logic (13, 18, 19). Pragmatic case finding (PCF) is a strategy based on clinical relevance, meaning the practitioner addresses alcohol when it is potentially relevant to the condition that the patient is presenting with, either as cause, complicating factor or due to increased vulnerability (13). Relevance-based approaches are more in line with GPs' skills and clinical reasoning and may potentially improve the widespread implementation of SBI (20, 21).

To date however, most studies on SBI have focused on universal, widespread screening; far fewer studies have explored pragmatic (targeted or relevance-based) strategies to identifying hazardous or harmful drinkers in health care settings (17, 18, 22). One study compared universal screening of all patients vs. targeted screening focused on clinical relevance. Targeted screening focused on five conditions, i.e., mental health, hypertension, gastrointestinal problems, minor injuries, and new patient registrations (23, 24). Even though targeted screening was more efficient in identifying patients, it also resulted in more missed cases of patients for whom brief intervention could have been beneficial. This points to a need for additional support and training required for GPs to effectively identify at-risk drinkers using relevance-based approaches, as using clinical judgement alone may result in missing some cases (17).

To our knowledge there are no existing training interventions to upskill GPs in the use of PCF, hence our study aims were twofold. First, an in-depth exploration of GPs' needs with regards to using PCF to addressing alcohol in general practice, and to more precisely unfold factors enabling an alcohol conversation based on this mind set. Second, to use these findings to inform the development of a tailored, theory-based intervention to strengthen GPs' ability to address alcohol and manage alcohol-related health problems based on clinical relevance.

## Materials and methods

### Design

#### Tailoring approach

Given our focus on supporting GPs with adopting new clinical practices we decided to apply a theory-informed intervention development process, focusing on behavior change. We considered three approaches with roots in behavioral and implementation sciences: Intervention Mapping (25, 26), the French model (27), and the Behavior Change Wheel [BCW; (28)]. We decided to apply the BCW because of its' focus on the nature of behavior as a starting point for change. It summarizes 19 frameworks of behavior change and includes the Capability, Opportunity, Motivation-Behavior (COM-B) model, which can be used to explore the determinants of GPs' behavior in context (28, 29). The COM-B model was recently used as a coding framework in a systematic review of factors influencing implementation of SBI, which identified the development of theory-based training programmes as one of the key research priorities (29).

#### Focus groups

This qualitative study, comprising focus groups and structured questionnaires, was undertaken in late 2018 and in 2019, in the cities of Stavanger and Oslo, Norway. Prior to performing the focus group interviews, aspects relating to the aims of the study were explored in individual conversations with GPs (*n* = 4) and patient representatives (*n* = 2) from a patient organization. One of the authors (TGL), a GP with expertise in alcohol related health problems and qualitative research, carried out all focus group interviews (30, 31). The groups were purposively sampled to provide a wide range of clinical experience and were consecutively invited aiming to maintain diversity in experience, age and gender. Focus groups provided a supportive environment that enabled the exploration of GPs' needs with regards to addressing alcohol in general practice. We applied an iterative approach whereby issues from the initial explorative process informed the interview guide for the first two focus group interviews, and the interview guide for the following groups were adjusted based on the discussions in the first groups. Participants were specifically invited to challenge and elaborate on these issues, as well as introduce new aspects. All focus groups were recorded and transcribed in full for analysis, and participant identifiers were removed.

#### Questionnaire

In parallel with the focus group interviews, we used a structured questionnaire based on the COM-B Self-Evaluation Questionnaire V1 (COM-B-Qu1), which is recommended for use during the BCW intervention development process (28). The questionnaire included 19 items to assess GPs' needs in terms of capabilities, opportunities, and motivation to address alcohol in consultations. We piloted the questionnaire in the focus groups after the interviews. GPs were asked to tick any relevant constructs and were encouraged to provide further detail in an open text box under each item. The final version of the questionnaire was developed based on the focus group interviews and the piloting and used at a mandatory seminar for young GPs on preventive strategies in primary care. All participants at the seminar were invited to reply, and they were specifically encouraged to give free text replies. About 10 min of the seminar was reserved for this, and the respondents completed the questionnaire in the seminar room. A completed checklist of the ‘Standards for Reporting Qualitative Research' (SRQR) can be found in Supplementary Table 1 (32).

Participants provided informed consent prior to participation in the study and received no compensation for taking part in the focus groups or for completing the questionnaire. The research protocol was approved by the Regional Committee for Medical and Health Research Ethics (Application No: 6,848) and by the data protection officer of Stavanger University Hospital (project ID 8/2020).

### Data analysis and approach to intervention development

**Figure 1:**
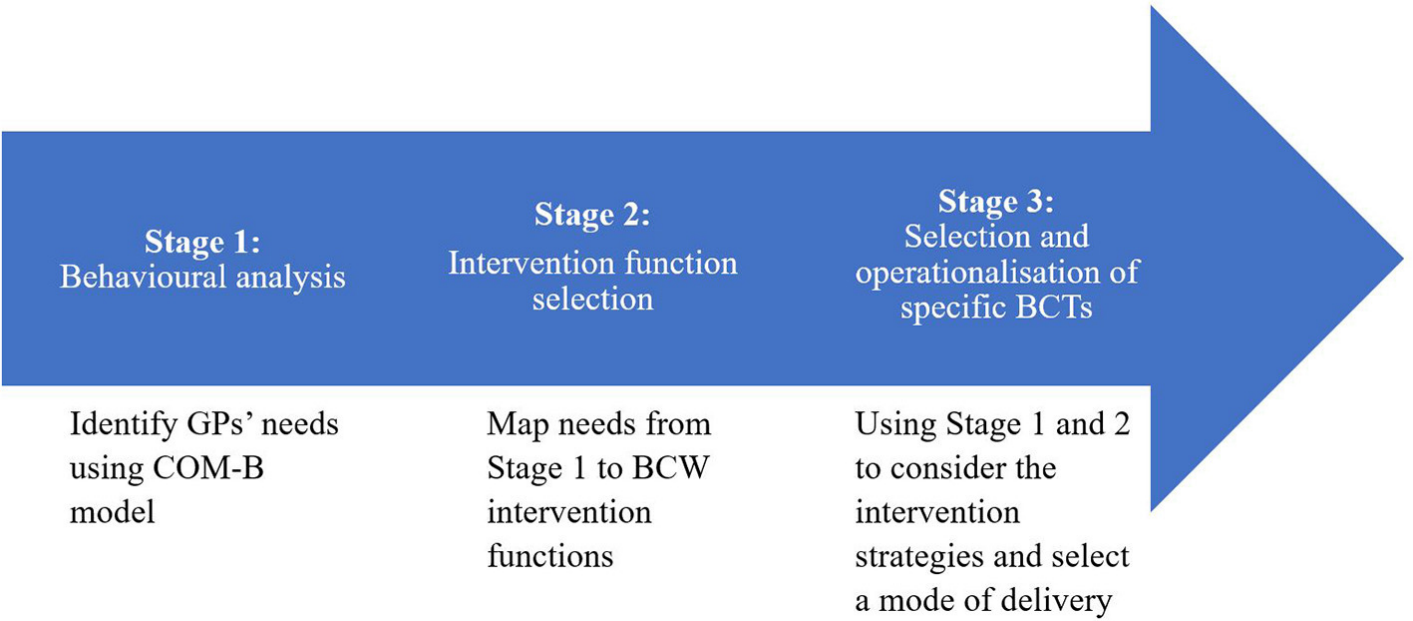
Behavior change wheel informed process for tailoring strategies to prospectively identified needs of general practitioners (22).

We used a three-stage approach to analyse data and develop our intervention. First, transcripts from the focus groups and structured questionnaires were behaviorally analyzed using the COM-B model to systematically determine what GPs needed to be able to effectively identify and address alcohol using *pragmatic case findings*. At this stage we considered existing research on barriers and facilitators of PCF and SBI more widely (21, 29), but decided that a more in-depth exploration using a robust implementation framework would be warranted. This is in line with tailored implementation approaches which highlight the need for identifying and prioritizing local determinants of practice, before matching strategies to address those determinants (33). Second, identified needs were mapped against BCW intervention functions. Third, and finally, we developed a suitable training intervention tailored to the findings (see Figure 1), with further detail on each stage provided below.

### Stage 1. Behavioral analysis

In the first stage we deductively analyzed the focus group interviews and COM-B-Qu1 responses to identify GPs' needs with regards to delivering *pragmatic case findings* for addressing alcohol in general practice (34). We used the COM-B model as an initial coding framework, mapping GPs' needs onto *physical* or *psychological capability, reflective* or *automatic motivation* and *social* or *physical opportunity*. Subsequently, an inductive thematic approach was applied to identify themes within COM-B sub-constructs. Two of the authors (SP and ATK) independently identified themes within each of the COM-B constructs (35). Next, the two authors integrated and finalized the themes through discussions with the wider group of co-authors. All analyses were conducted according to the standard procedures of rigorous qualitative analysis (36).

### Stage 2. Intervention function selection

In the second stage we selected the most appropriate intervention functions by mapping the needs identified in the behavioral analysis onto the published BCW linkage matrix (37). We considered each of the intervention functions that could be relevant to our data and next used the APEASE (affordability, practicability, effectiveness, and cost effectiveness, acceptability, side effects/safety and equity) criteria to prioritize the most relevant functions of our intervention (28).

### Stage 3. Selection and operationalisation of specific behavior change techniques

Within each of the identified intervention functions we selected specific behavior change techniques (BCTs) that best served and operationalised the intervention functions. There are a large number of BCTs (i.e., 93) that have been summarized in the behavior change technique taxonomy (BCTTv1) (38). We selected those techniques that were most frequently used with our selected intervention functions, based on links previously identified between the BCW and the BCTTv1 (37). Next, we prioritized the most appropriate BCTs by using the APEASE criteria. Following several group discussions and expert consultations, we selected 11 BCTs to address the determinants identified in the needs analysis. We used the Template for Intervention Description and Replication (TIDieR) Checklist to support the operationalisation of the prioritized BCTs (see Supplementary Table 2).

## Results

### Participant characteristics

A total of four GP focus groups were conducted, lasting between 45 and 90 min: one with five GPs (*n* = 3 female, 2 male) from a GP clinic in Stavanger; one with seven GPs (*n* = 4 female, 3 male) from a GP clinic in Oslo, and two separate focus groups with continuous medical education (CME) groups in Stavanger with 13 GPs (5 female, 8 male) (Table 1). The participants had a wide range of clinical experience, from 2 to 3 years to more than three decades in clinical practice, with a reasonable gender balance. Twenty out of the 25 GPs were certified and 5 were working toward their certification.

**Table 1:** Demographics of participants in the focus group interviews in two GP clinics and two peer groups (CME-groups).

**Age groups**	**Female**	**Male**	**Total**
30–39	1	3	**4**
40–49	3	3	**6**
50–59	5	4	**9**
60–69	4	2	**6**
**Total**	**13**	**12**	**25**

A paper version of the questionnaire was handed out to physicians specializing in family medicine during a mandatory seminar on preventative medicine in general practice in Stavanger in September 2019 (*n* = 68, female 40, male 28). This group was chosen due to slightly more older participants than younger in the focus groups. Of the 68 participants in the mandatory seminar, 55 returned a completed questionnaire (Table 2).

**Table 2:** Demographics of participants invited to complete COM-B questionnaire during a mandatory seminar on preventive medicine for physicians becoming certified GPs.

**Age groups**	**Female**	**Male**	**Total**
20–29	4	1	**5**
30–39	24	18	**42**
40–49	10	5	**15**
50–59	2	4	**6**
Total	**40**	**28**	**68**
Completed questionnaires			**55**

### Stage 1. Behavioral analysis

#### Capability (psychological)

GPs highlighted the need for effective strategies for opening a conversation around a patient's alcohol consumption. It was acknowledged that alcohol consumption may be a sensitive topic (e.g., due to embarrassment) that the patient may not bring up on their own. In such situations, most GPs agreed that it was their responsibility to create a non-judgemental atmosphere within which a patient felt comfortable to speak about alcohol:

“For most doctors and patients, it is difficult to start the conversation. Patients often don't want to show that they have a drinking problem. Then it's the doctor's responsibility to open up to the topic in a comfortable way.” [COM B, CME seminar]

Bringing up alcohol as part of general or universal screening was perceived as a less useful approach by some GPs as they thought patients would not see the relevance to their health:

“It is not so desirable to get help with this, that's also a point. There's no business in structured case finding. Because people, basically, with a few exceptions, are uninterested.” [Peer group 1, GP2, male]

Instead, most GPs felt that it would be better to frame the alcohol conversation around health problems that patients presented with, such as sleeping problems, depression or hypertension. To support this approach, GPs highlighted the need for accurate information on the relationship between alcohol, health problems and drug interactions. Further they expressed the need for this knowledge to be based on accurate evidence from clinical research:

“Because we need knowledge, we cannot use thoughts of what we think would be good, to implement a change that for some feels very invasive. It will backfire if it [the knowledge] isn't founded properly.” [Peer group 1, GP2, male]

GPs suggested there was a need for specific strategies for dealing with sensitive situations. In part, this was because of the stigma that is still attached to heavy drinking. However, participants also raised concerns about the adverse impact of the mandated function for Norwegian GPs to consider whether the patient's health status, including alcohol consumption, might affect the patients' “right to drive”. Even though some presented examples where this helped motivate the patient to cut down on alcohol, several participants had experienced that patients may withhold information about their alcohol consumption because they were worried about losing their driver's license:

“I had a woman once asking me about liver tests. So I asked her why. Because she wondered whether she was drinking too much. Then she said ‘I really didn't want to ask you, because I know you can revoke my driver's license'.” [Peer group 2, GP2, female]

#### Opportunity (social)

Some GPs thought it was necessary to better prepare patients to have a conversation about alcohol-related health problems. There was a perception that preparing patients in advance would make them feel more comfortable and thus enable a more constructive conversation about their drinking to take place:

“I think it consists of several components. In order for us to get this to be robust, it must both be an invitation to talk about alcohol, and the patient needs to be primed that this is something the doctor is actually talking about.” [Peer group 1, GP2, male]

Many GPs expressed the need for better knowledge of and collaboration with specialized services. They acknowledged that they felt demotivated to bring up the topic of alcohol without sufficient knowledge of specialized services to refer to:

“[Need an] outline of the institutions, patients often wait for a long time and want help fast when they have acknowledged the problem.” [COM-B, CME seminar]

Some GPs also expressed a need for a ‘shared culture' of addressing alcohol within their clinic. A ‘shared culture' was viewed as one that provided opportunities for GPs to exchange ideas about how to address alcohol most optimally within their clinic:

“It would be an advantage with collegial agreement [on addressing alcohol]. Also in the case of absence, so my colleagues can continue with follow-up of my patients.” [COM-B, CME-seminar]

#### Opportunity (physical)

GPs highlighted the need for simple, accessible and relevant materials that they could use to provide patients with information about alcohol related health problems. GPs also saw the potential of such materials to support a structured approach to follow-up with the patient after the consultation. Participants expressed the need for both physical brochures as well as digital resources that are easy to use and not too demanding:

“I would think it would be nice if there was some more written info relevant for the patients, because this is incredibly good for them (to have).” [Peer group 2, GP3, female]

Some GPs also emphasized the need for sufficient time to talk about alcohol, given the topic's sensitive and personal nature. And some suggested to add alcohol and related health problems as an item to the agenda for the consultation:

“And then I think you have to put it on the agenda as a topic that we must address today. For some reason. There may be a problem related to the issue the patient is coming for. Or that we have taken a blood test which shows that it may be relevant for example. Because they took a series of blood tests. So, I think time is something that must be here.” [Peer group 2, GP1, male]

#### Motivation (automatic)

Some GPs talked about the need to get into a routine of asking about alcohol when relevant. They highlighted the need to connect the alcohol conversation to other routine situations such as general health checks, blood tests, drug reviews, before surgery or pregnancy:

“But if we had a routine asking how much you drink when taking these [blood] tests. Obviously, it is not always relevant, but if there were some kind of automaticity in it, it would be good.” [Peer group 1, GP4, male]

A list of when it is relevant to talk about alcohol, including various related health problems, was seen as useful by participants. GPs suggested that having a list of relevant conditions could serve as a reminder that keeps alcohol as a possible cause in the back of their mind.

### Stage 2. Intervention function selection

#### Capability

GPs voiced their concerns over their lack of confidence and skills about opening up the conversation, offering accurate information, and dealing with challenging situations. To address these needs, the intervention functions *Education, Training, Enablement*, and *Modeling* were chosen to provide evidence-based knowledge and practical tools, while enabling behavioral practice and providing verbal persuasion about their capabilities to address alcohol related health problems.

#### Opportunity

GPs highlighted the need for enabling opportunities to address alcohol-related health problems by preparing patients for the conversation, facilitating collaboration with specialized services, providing follow-up support and creating a shared culture. To address these needs the intervention functions *Environmental Restructuring, Enablement* and *Training* were chosen to facilitate patient activation, establish collaboration with specialized services and train GPs in the use of an e-health intervention to support follow-up.

#### Motivation

Most GPs expressed a need for getting into a routine for asking about alcohol when a patient with a related health problem presents. The intervention function *Education* was chosen to teach GPs about pragmatic case finding as a semi-structured strategy for identifying at-risk drinkers based on clinical relevance. The intervention function *Training* was chosen to support GPs with behavioral practice and habit formation.

### Stage 3. Selection and operationalisation of specific behavior change strategies

#### The final intervention

**Figure 2:**
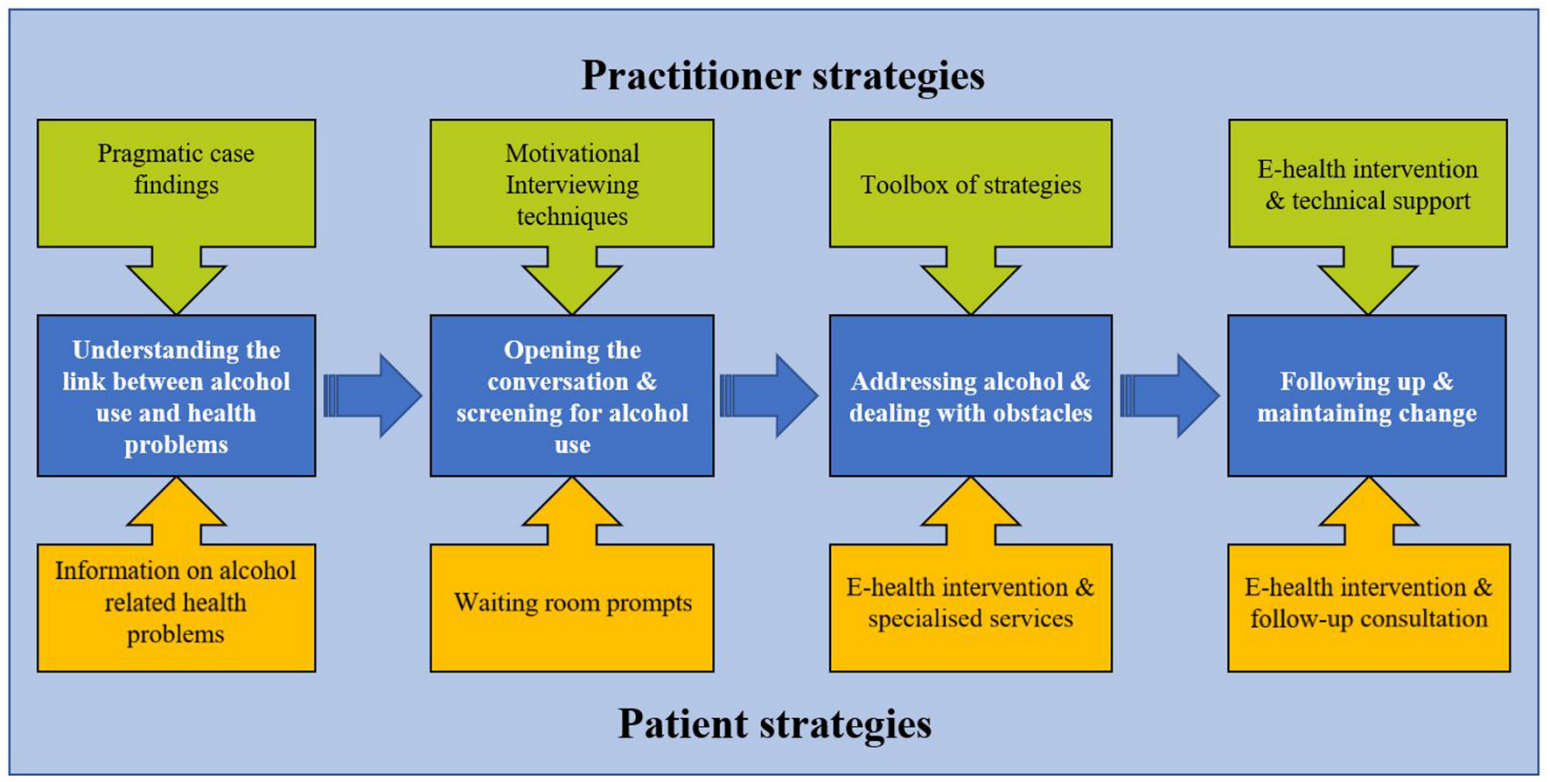
Tailored strategies to support general practitioners, practice staff and patients in addressing alcohol-related health problems using pragmatic case finding.

The final training intervention consists of four clinical seminars (3–4 h in length) to equip GPs and other staff members with the skills and tools to implement a pragmatic approach to addressing alcohol-related health problems in routine care. Each seminar is led by an experienced facilitator who has expertise in the covered topic areas including *addiction, motivational interviewing, e-health*, and *behavior change*, and with extensive experience from general practice. Each seminar will use a range of delivery modes including *presentations, group discussions, role plays, case examples* and *homework exercises*. Table 3 presents an overview of the identified needs, matched intervention functions, BCTs and their modes of delivery in the final seminars. The link between all training strategies (practitioner and patient) and identified needs is displayed in Figure 2. The completed TIDieR checklist is presented in Supplementary Table 3. A full programme for the seminar series can be found in Supplementary Table 4.

**Table 3:** Matrix of links between COM-B components, identified needs, intervention functions, behavior change strategies, and intervention content.

**COM-B component**	**Needs identified**	**Selected intervention function**	**Selected behavior change technique (Code in BCTTv1)**	**Operationalisation of behavior change technique**
*Capability (psychological)*	Need for effective communication strategies	- Education - Training -Enablement -Modeling	- Instructions on how to perform a behavior - Demonstration of the behavior - Behavioral practice/ rehearsal - Habit formation	Training in **communication techniques** (e.g. motivational interviewing) for opening up a conversation around alcohol. Training in a **toolbox of intervention strategies** (e.g., biofeedback) for supporting patients with reducing their alcohol consumption. **Homework** will be assigned to support behavioral rehearsal of the techniques and strategies in clinical practice.
	Need for accurate information on the relationship between alcohol, health problems and drug interaction	- Education	- Credible source - Information about health consequences	An experienced general practitioner will present practice staff with **evidence-based information** on the association between alcohol consumption and health problems.
	Need for strategies for dealing with challenging situations	-Enablement	-Problem solving	GPs will be prompted to brainstorm a range of potentially **challenging situation** (e.g. ethical and practical problems regarding drivers' license and family issues) and ways of dealing with these situations (e.g., using their own patient scenarios)
*Opportunity (social)*	Need for preparing patients for a conversation about alcohol	- Environmental restructuring	-Prompts/cues -Information about health consequences	**Posters** will be displayed in the practice waiting rooms to prompt patients to talk to their GP if they are experiencing potentially alcohol-related health problems.
	Need for better cooperation with specialized services	-Environmental restructuring	- Restructuring the social environment	An **interactive discussion** will address referral to and collaboration with specialized services. Representatives from relevant services (community based, user organization, and specialized services) will discuss scenarios and collaboration.
	Need for a shared practice culture for addressing alcohol	-Environmental restructuring	- Restructuring the social environment	The seminar is designed to facilitate a **shared culture** in general and on this specific topic, by addressing the practice as such, aiming to include all GPs in the practice, and including staff in all sessions.
*Opportunity (physical)*	Need for relevant and accessible materials	-Education -Training	-Instructions on how to perform a behavior	GPs will receive a **website link with relevant resources**. The resources will be developed during the first phase (first two surgeries) of the feasibility study, as a repository of the seminar materials (e.g., list of videos), including links to relevant resources (e.g., papers and relevant screening tools).
	Need for follow-up support	-Enablement	-Instructions on how to perform a behavior -Demonstration of the behavior - Behavioral practice/ rehearsal - Problem solving	An e-health expert will train all practice staff in the use of **‘Endre'** a **self-administered e-health intervention** to address alcohol-related health problems (e.g., sleeping problems, weight gain, hypertension, and mental health problems) between consultations.
*Motivation (automatic)*	Need for getting into a routine of asking about alcohol when relevant	-Education -Training	-Self-monitoring of behavior - Action planning	GPs will be introduced to **pragmatic case finding (PCF)**, a semi-structured strategy for identifying at-risk drinkers based on clinical relevance. GPs will be informed about the role of alcohol in various common health conditions and treatments, a **list of clinical relevance**. GPs will be prompted to use this list to help them form a **specific plan for when, where and how** they will use PCF as a strategy to identify alcohol-related health problems.

Taught tools and strategies include *communication techniques, pragmatic case finding, toolbox of strategies*, and an *e-health intervention. Communication techniques* include strategies from Motivational Interviewing (MI), such as expressing empathy through reflective listening (39). *Pragmatic case finding* is a semi-structured strategy for identifying at risk-drinkers based on clinical relevance (13). The *toolbox of intervention strategies* includes behavior change strategies, biofeedback using specific and non-specific biomarkers for alcohol, medication, and options for referral and / or collaboration with community based and specialized services. The *e-health intervention*, called “*Endre*” (a male first name in Norwegian, and also Norwegian for “change”), is a self-administered intervention to address alcohol-related health problems between consultations. Endre was initially developed as a smoking cessation program, and the development of the present version was based on the initial intervention (40, 41). An e-health expert (one of the developers) will provide on-demand technical support on *Endre via* phone or email. Endre addressed GPs' need for an accessible electronic resource which could be used by patients between sessions to support their reduction of alcohol consumption. The systematic development of Endre will be reported elsewhere (40, 41).

To facilitate implementation from a patient perspective, there will be *waiting room prompts* (posters) and *information leaflets* that will encourage patients to speak to their GP if they are experiencing alcohol-related health problems.

## Discussion

The purpose of this study was an in-depth exploration of GPs' needs with regards to using *pragmatic case findings* as an approach to addressing alcohol, and to tailor implementation strategies to address the identified needs. Using the Behavior Change Wheel approach, we identified four areas that required additional support, including: understanding the link between alcohol use and health problems; opening up the conversation and screening for alcohol use; addressing alcohol and dealing with obstacles; and following-up and maintaining change. To address these needs, we developed a four-session interactive training intervention, utilizing evidence-based behavior change strategies, to equip GPs and other staff members with the skills and tools to implement a pragmatic approach to addressing alcohol-related health problems in primary care.

### Comparisons with existing literature

Previous research has identified a lack of tailored training and support as one of the main barriers to integrating and sustaining SBI in general practice. Existing evaluations of training interventions aimed at increasing screening for alcohol consumption have yielded modest effects (42). One reason for limited effectiveness is a lack of multicomponent interventions which address GPs' needs in terms of capabilities, opportunities, and motivations. Previous interventions aimed to implement SBI have addressed only a limited number of barriers and relied on a one-size-fits-all approach (43). Our study highlights the need to tailor SBI to local needs and capacities to achieve their sustained adoption in primary care (44, 45). Such an approach is in line with several emerging studies in the field. For example, one study uses a structured implementation framework to tailor clinical materials and training to provider and user needs (46). Another study aims to use the BCW and TDF to tailor training and community mobilization to improve the uptake of SBI (47). Our study is novel in that the tailored training intervention was developed to implement a pragmatic, relevance-based approach to SBI.

In line with a previous qualitative study, also from Norway, we found a need to better integrate SBI within GPs' existing working routines (15), allowing them to focus alcohol-related discussions on patients presenting with issues that may be affected by alcohol consumption (13). Our findings suggest that such pragmatic, relevance-based approaches may be more sustainable than universal screening, as they fit more closely with GPs' existing skill sets and clinical preferences. Importantly, the aim of such strategies is not primarily to identify patients drinking above recommended limits or patients with alcohol use disorders, but to identify whether alcohol may affect a patients' clinical problem. This reflects the results of a Delphi study conducted in the Netherlands that found strong practitioner support for asking about alcohol when clinically relevant, based on physical, social or psychological symptoms (48). The current training intervention was designed to address this need by providing GPs with education and tools that help them recognize at risk groups and common clinical conditions frequently affected by alcohol use. Action planning was incorporated to help GPs integrate PCF in their routine by getting them to formulate specific plans for when, where and how they will use PCF in their own practice (49, 50). There is good evidence showing that action planning is effective for supporting behavior change in patients and the public (51), and evidence is starting to emerge for their effectiveness in changing practitioner behavior (52).

Our findings highlight the need for creating a safe environment and trusting relationship between patients and GPs to facilitate conversations around alcohol use. This reflects existing evidence, including the previously mentioned qualitative study in Norway that showed that GPs are often worried about the negative impact that raising alcohol with the patient might have on the patient-practitioner relationship (15). This lends further support for the pragmatic, symptom-based approach taken in this study, as it helps to more directly link alcohol consumption to the patients' agenda (13). Another strategy we have matched to address this need is training GPs in Motivational Interviewing (MI), which is a directive, patient-centered counseling style that can promote behavior change. Motivational interviewing is seen as a non-confrontational strategy to addressing alcohol consumption, and one systematic review found brief MI to be effective for reducing alcohol consumption (39, 53). Collaborative problem solving was included to allow GPs to share challenging situations from their practice and brainstorm, together with communication experts, ways of dealing with these situations. Overall, the training is designed to be interactive, using a variety of delivery modes, to facilitate collaboration and knowledge exchange that will enable integration of pragmatic approaches to addressing alcohol in primary care.

### Strengths and limitations

This study applied an in-depth qualitative, theory-based approach to identifying local determinants of SBI to match evidence-based behavior change strategies to address the identified determinants. Within the implementation literature, there is widespread agreement that intervention tailored to local determinants of practice are more effective than more generic interventions (33). For example, the quality improvement literature highlights that the effectiveness of implementation strategies may depend on how well they are adapted to the services' circumstances, and how they can co-exist with local facilitators in a specific service (54). The unique contribution of our study is the application of the Behavior Change Wheel as a theoretical framework for matching GPs' needs with evidence-based techniques to facilitate clinical behavior change. Another strength of this study is that it investigated the implementation of a pragmatic (or targeted) approach to addressing alcohol that fits more closely with the primary care context and which builds on the skills that GPs have and use in practice. Previous research has shown that pragmatic case finding is a relevant and viable approach for addressing alcohol and seen as a more implementable alternative to universal approaches (13). Although we know that relevance-based approaches to SBI are more acceptable and efficient than universal approaches, there is a risk that patients who may benefit from brief intervention are missed. This is because GPs lack the relevant knowledge on how increased alcohol consumption influences other health conditions. They also often lack tools and skills to open up the conversation and intervene when relevant. If found effective, our training intervention will provide GPs with the necessary knowledge and skills to more accurately identify at risk drinkers and intervene when clinically relevant.

One limitation of this study is that the designed implementation intervention was tailored mostly around GPs' needs and did not explore patients' views and preferences in as much detail. However, we did actively engage with two patient representatives throughout the design process and incorporated their feedback where possible. For example, GPs suggested several strategies that would facilitate patient involvement, including waiting room prompts and information, and a digital intervention to support patients between consultations. Both patient representatives agreed with the suggested strategies and helped in the design process. Another limitation is the length of the designed training intervention (four sessions), which was chosen to enable GPs to fulfill the Norwegian requirement for qualification and requalification, which includes at least five clinical seminars of at least 15 h each 5 year period. However, the four-step approach including practitioner and patient strategies presented in Figure 2 could easily be delivered in shorter, less time and resource intensive formats (e.g., using E-learning technology).

### Implications for research

This intervention was developed for a future trial to test the feasibility of the clinical intervention (PCF) and the implementation intervention (the described training intervention). The clinical intervention consists of two components, namely PCF and a digital self-administered intervention (Endre) for use for patients between consultations (40, 41). GPs in the feasibility study will complete the Determinants of Implementation Behavior Questionnaire (DIBQ) at the start and the end of the study to determine whether the developed training intervention is effective in addressing the identified determinants of practice (55). The feasibility trial will focus on acceptability, demand, implementation, and practicability of the interventions and indicate whether a full-scale RCT is warranted.

Future research could explore alternative modes of delivering the training intervention such as E-learning technology. E-learning technology could enable the tailoring of training content to the needs of the individual practitioner and facilitate interactions with others. This technology would be especially advantageous during times when physical interactions are not possible (e.g., during a pandemic) or in countries where GPs have limited time or financial resources for continuous professional development. Internet-based learning has been shown to be at least as effective as traditional learning methods as it enables GPs to access learning content at a time and place that is convenient to them (56–58).

## Conclusion

Despite of their proven effectiveness, implementation of alcohol screening and brief behavioral interventions (SBIs) in routine primary care remains difficult. Universal screening is resource-intensive and at odds with general practitioners' (GPs) perceived role. Drawing on the Behavior Change Wheel approach, this article identifies GPs' needs with regards to forming a routine of adopting SBI practices. This work has highlighted GPs' perspectives on barriers and facilitators to addressing alcohol and provided in-depth knowledge on specific requirements for improving practice. Training GPs in pragmatic case finding (PCF) as an alternative to screening should include strategies to prepare and open up the conversation, specific knowledge on how alcohol may be related to the health problem, and strategies to support change and to utilize other resources when needed. PCF builds on practitioners' existing clinical skill set, whilst raising awareness of the often-overlooked connections between many health problems and alcohol consumption. This article describes the PCF approach and outlines a tailored intervention to equip GPs with the knowledge and skills needed to adopt PCF in their clinical settings.

## Data availability statement

The datasets presented in this article are not readily available because this would likely compromise participants' anonymity. Some descriptive data may be available from the corresponding author on reasonable request. Requests to access the datasets should be directed to sebastian.potthoff@northumbria.ac.uk.

## Ethics statement

The studies involving human participants were reviewed and approved by Regional Committee for Medical and Health Research Ethics (Application No: 6848) and by the data protection officer of Stavanger University Hospital (project ID 8/2020). The patients/participants provided their written informed consent to participate in this study.

## Author contributions

SP: conceptualization, methodology, formal analysis, writing—original draft, and writing—review and editing. AO: conceptualization, methodology, and writing—review and editing. ATK: conceptualization, methodology, formal analysis, data collection, writing—review and editing. HB: conceptualization, methodology, writing—review and editing. TGL: conceptualization, methodology, formal analysis, data collection, and writing—review and editing.

## Funding

This study did not have any specific funding but was conducted as part of the authors' regular employment. SP is a member of the NIHR Applied Research Collaboration North East and North Cumbria (NIHR200173). AO is funded by a National Institute for Health Research (NIHR), Advanced Fellowship (grant reference: NIHR300616). The views expressed are those of the author (s) and not necessarily those of the NIHR or the Department of Health and Social Care.

## Conflict of interest

The authors declare that the research was conducted in the absence of any commercial or financial relationships that could be construed as a potential conflict of interest.

## Publisher's note

All claims expressed in this article are solely those of the authors and do not necessarily represent those of their affiliated organizations, or those of the publisher, the editors and the reviewers. Any product that may be evaluated in this article, or claim that may be made by its manufacturer, is not guaranteed or endorsed by the publisher.
