# Supplementary material for: Pragmatic approaches for addressing alcohol in general practice: Development of a tailored implementation intervention
Source: Front Health Serv. 2022 Nov 17;2:940383. doi: 10.3389/frhs.2022.940383 (PMC10012791; doi:10.3389/frhs.2022.940383)
Supplement: Supplementary file 1 [file Data_Sheet_1.PDF]

frontiers

Supplementary Material

Supplementary Table 1. Completed 'Standards for Reporting Qualitative Research' (SRQR) checklist.

**Standards for Reporting Qualitative Research (SRQR)**	
http://www.equator-network.org/reporting-guidelines/srqr/<br><br>O'Brien BC, Harris IB, Beckman TJ, Reed DA, Cook DA. Standards for reporting qualitative research: a synthesis of recommendations. Acad Med. 2014 Sep;89(9):1245-51. doi: 10.1097/ACM.0000000000000388. PMID: 24979285.	
	**Page numbers**

Title and abstract

**Title** - Concise description of the nature and topic of the study Identifying the study as qualitative or indicating the approach (e.g., ethnography, grounded theory) or data collection methods (e.g., interview, focus group) is recommended	1
**Abstract** - Summary of key elements of the study using the abstract format of the intended publication; typically includes background, purpose, methods, results, and conclusions	2

Introduction

**Problem formulation** - Description and significance of the problem/phenomenon studied; review of relevant theory and empirical work; problem statement	3-4
**Purpose or research question** - Purpose of the study and specific objectives or questions	4

Methods

**Qualitative approach and research paradigm** - Qualitative approach (e.g., ethnography, grounded theory, case study, phenomenology, narrative research) and guiding theory if appropriate; identifying the research paradigm (e.g., postpositivist, constructivist/ interpretivist) is also recommended; rationale**	4
**Researcher characteristics and reflexivity** - Researchers' characteristics that may influence the research, including personal attributes, qualifications/experience, relationship with participants, assumptions, and/or presuppositions; potential or actual interaction between researchers' characteristics and the research questions, approach, methods, results, and/or transferability	6
**Context** - Setting/site and salient contextual factors; rationale	5-6
**Sampling strategy** - How and why research participants, documents, or events were selected; criteria for deciding when no further sampling was necessary (e.g., sampling saturation); rationale	5-6
**Ethical issues pertaining to human subjects** - Documentation of approval by an appropriate ethics review board and participant consent, or explanation for lack thereof; other confidentiality and data security issues	14
**Data collection methods** - Types of data collected; details of data collection procedures including (as appropriate) start and stop dates of data collection and analysis, iterative process, triangulation of sources/methods, and modification of procedures in response to evolving study findings; rationale	4
**Data collection instruments and technologies** - Description of instruments (e.g., interview guides, questionnaires) and devices (e.g., audio recorders) used for data collection; if/how the instrument(s) changed over the course of the study	4
**Units of study** - Number and relevant characteristics of participants, documents, or events included in the study; level of participation (could be reported in results)	5-6
**Data processing** - Methods for processing data prior to and during analysis, including transcription, data entry, data management and security, verification of data integrity, data coding, and anonymization/de-identification of excerpts	5

**Data analysis** - Process by which inferences, themes, etc., were identified and developed, including the researchers involved in data analysis; usually references a specific paradigm or approach; rationale	5
**Techniques to enhance trustworthiness** - Techniques to enhance trustworthiness and credibility of data analysis (e.g., member checking, audit trail, triangulation); rationale	5

Results/findings

**Synthesis and interpretation** - Main findings (e.g., interpretations, inferences, and themes); might include development of a theory or model, or integration with prior research or theory	9-10
**Links to empirical data** - Evidence (e.g., quotes, field notes, text excerpts, photographs) to substantiate analytic findings	5-9

Discussion

**Integration with prior work, implications, transferability, and contribution(s) to the field** - Short summary of main findings; explanation of how findings and conclusions connect to, support, elaborate on, or challenge conclusions of earlier scholarship; discussion of scope of application/generalizability; identification of unique contribution(s) to scholarship in a discipline or field	10-13
**Limitations** - Trustworthiness and limitations of findings	11

Other

**Conflicts of interest** - Potential sources of influence or perceived influence on study conduct and conclusions; how these were managed	14
**Funding** - Sources of funding and other support; role of funders in data collection, interpretation, and reporting	14

Supplementary Table 2. Completed Template for Intervention Description and Replication (TIDieR) for the tailored training intervention

TIDieR
Template for Intervention Description and Replication

The TIDieR (Template for Intervention Description and Replication) Checklist*: Information to include when describing an intervention and the location of the information

No.	Item	Intervention description
**1.**	**BRIEF NAME**<br><br>Provide the name or a phrase that describes the intervention.	Clinical topic seminar series on pragmatic alcohol screening and brief intervention for GPs
**2.**	**WHY**<br><br>Describe any rationale, theory, or goal of the elements essential to the intervention.	1. In spite of proven effectiveness for brief interventions, GPs are frequently not addressing alcohol consumption when it is clinically relevant.<br>2. Implementation of universal screening for risky/harmful drinking in general practice remains problematic.<br>3. We propose *pragmatic case finding (PCF)* as a semi-structured identification strategy based on clinical relevance. PCF may potentially offer a more feasible and adaptable alternative to universal screening strategies and may fit more closely with GPs' existing skill sets.<br>4. GPs lack the necessary skills and/or tools for effectively managing risky/ harmful drinking and to assist patients with navigating digital interventions in general practice.<br>5. E-health interventions have some effect, but few studies have examined the potential added value of blended interventions in general practice.<br>6. GPs' mandated control function regarding driver's license, to ensure that drivers possess the necessary health status to obtain or maintain a right to drive a car. The GPs' responsibilities in this regard have been increased lately, especially on alcohol and drug problems, affecting both patients' and GPs' inclination to discuss alcohol consumption.<br>7. To become or to remain a specialist in family medicine in Norway every GP has to attend at least five different certified seminars on clinical topics per five years, of at least 15 hrs each, and at least 100 hrs in total. While Norwegian Medical Union normally reimburses travel costs and accommodation, these seminars are quite costly because of the fees and the need to leave the clinic. Most GPs in Norway are independent contractors.
**3.**	**WHAT**<br><br>Materials: Describe any physical or informational materials used in the	• *List of clinical relevance.* Clinical conditions and situations where there is evidence that alcohol may affect the health of the patient. Alcohol may precipitate or complicate many health problems. This will be a list of key conditions and contexts including clinical examples. The list will be used throughout the seminars to raise GPs' awareness of

	intervention, including those provided to participants or used in intervention delivery or in training of intervention providers. Provide information on where the materials can be accessed (e.g. online appendix, URL).	alcohol in relation to these conditions. A laminated prompt sheet with the list will be provided for the GPs.<br>• *Patient scenarios.* Given in the clinically focused presentations (ppt, stories), in all sessions. Conveying key learning points in relation to identifying and treating alcohol related health problems. For example, to demonstrate the role of alcohol in the development and maintenance of relevant health problems, or how GPs can initiate a conversation around alcohol and then follow-up on the topic in the long-term.<br>• *Strategies for preparing for and opening up a conversation around alcohol.* Practical examples and discussions on how to open up a conversation with the patient around alcohol and health. GPs will be prompted to share and discuss their own patient scenarios. Examples added to the website with relevant resources (see later).<br>• *Toolbox of intervention strategies.* Including motivational strategies (MI), volitional strategies (action and coping planning), non-medication based intervention strategies (e.g. physical activity, support groups), biofeedback using specific and non-specific biomarkers for alcohol (e.g. GGT, CDT, PEth), medication (e.g. naltrexone, accamprosate, nalmefen), and options for referral/ collaboration with specialist services<br>• *E-health intervention.* Web-based application focusing on alcohol and health. Addressing how alcohol affects other related health problems (e.g. sleeping problems, weight gain, hypertension, and mental health problems). Includes monitoring and facilitating change in alcohol consumption and the related health problems. Intervention strategies are based on motivational interviewing techniques. The e-health intervention aims to assist the patients in preparing for follow-up consultations with their GP, by working on the potential link between their health problems and alcohol use.<br>• *Brief manual and flowchart for setting up the digital intervention.* Instructions on how to set up a new user, how to register baseline data for the feasibility study, and how to use *Endre,* for GPs and staff.<br>• *Website with relevant resources.* Will be developed during the first phase (first two surgeries) of the feasibility study, as a repository of the seminar materials (e.g., lists and videos), including links to relevant resources (e.g. papers and relevant screening tools).<br>• *Equipment.* Digital screen and/or projector for presentations/introductions, and taking notes from discussions along the way (white board, flip chart, sticky notes etc).<br>• *Templates and/or brief informational sheet*. For the homework assignments.
**4.**	Procedures: Describe each of the procedures, activities, and/or processes used in the intervention, including any enabling or support activities.	*1 (3 hours), GPs and staff (nurses, lab staff, admin staff):*<br><br>• 5.1 Information about health consequences; the relationship between alcohol, health and illness, with discussion (Why is it important?) (*GP)*<br>• 4.1 Instruction of how (and when) to perform, by introduction to PCF and the list of clinical relevance, with discussion (*GP)*<br>• Introduction to the digital intervention (Endre-Change) (*E-health expert)*<br>• Examples on how to implement *Endre* within the organisation, initial discussion on local adaptation (Tailoring)

		• Homework relating to *'list of clinical relevance',* counting consultations on topics from the list, and on local adaptation of how to implement *Endre*. *(GP and e-health expert)*<br>• Overview of the feasibility study (e.g. data collection)<br>*2 (4 hours) – GPs (other staff participate on the first point):*<br>• Endre training including Q&As *(E-health expert)*<br>• Discussion on the 'counting homework'. Reflecting and sharing experiences.<br>• *Alcohol – easier than you think.* Brief discussion on the 'counting homework'. In-depth education and training on PCF. Practical strategies for the GP, with clinical examples and evidence (Tailoring—focusing on conditions they are most interested in). GP's toolbox. 'White periods', discussion of clinical experiences. *(GP and/or addiction specialist)*<br>• Brief group discussion on how to open up the topic of alcohol with the patient. Groups feeding back their strategies. Trainer picking up points and expanding on them where possible (using *'strategies for preparing for and opening up a conversation around alcohol*). *(GP and/or MI-expert)*<br>• *Strategies for preparing for and opening up a conversation around alcohol,* presenting examples - homework<br>*3 (4 hours) – GPs and staff:*<br>• Endre discussion, including Q&A's focused on the problems and challenges experienced by users (patients, GPs and staff). *(E-health expert)*<br>• Alcohol – easier than you think part 2. Follow-up on session 2. Brief discussion on '---*opening up a conversation around alcohol* homework' – sharing experiences and discussing strategies, aiming to identify good examples. PCF and toolbox (*GP and/or addiction specialist)*<br>• MI-based teaching and training on motivation and change (essential part of the toolbox) – including role play. MI strategies, including goal setting, action planning, and alternative coping strategies (stressful life events) *(MI-expert)*<br>• *Practicing toolbox strategies* - homework. Taking notes on which strategies they use, how often, and how does it work.<br>*4 (4 hours) - GPs:*<br>• Endre – trouble shooting and Q&A. Thoughts on whether *Endre* can be a useful tool.<br>• Discussion homework, which strategies have they used, how often have they used them, and what are their experiences. White board and/or sticky notes.<br>• Ethical and practical problems regarding drivers' license and family issues – brief presentation before discussion *(GP)*<br>• Addictive drugs – brief presentation before discussion *(GP or other psychiatrist/addiction specialist)*

		• Referral to and collaboration with primary care and specialist services and other local alternatives
5.	**WHO PROVIDED**<br><br>For each category of intervention provider (e.g. psychologist, nursing assistant), describe their expertise, background and any specific training given.	• Expert (GP) on alcohol and related health problems, including PCF<br>• Addiction specialist<br>• Expert in MI from local competency centre<br>• Community level alcohol and life style specialist<br>• Expert on brief interventions in primary healthcare<br>• Expert on e-health interventions
6.	**HOW**<br><br>Describe the modes of delivery (e.g. face-to-face or by some other mechanism, such as internet or telephone) of the intervention and whether it was provided individually or in a group.	• Face-to-face seminars<br>• Website with resources<br>• On demand technical support on Endre via phone or email, delivered by e-health expert or MI-expert<br>• Feedback form for GPs and patients (on addressing alcohol and on Endre)
7.	**WHERE**<br><br>Describe the type(s) of location(s) where the intervention occurred, including any necessary infrastructure or relevant features.	• GP practices<br>• Digital screen and/or projector<br>• White board, flip chart, sticky notes etc.<br>• Internet access
8.	**WHEN and HOW MUCH**<br><br>Describe the number of times the intervention was delivered and over what period of time including the number of sessions, their	• A certified 15 hrs clinical topics seminar, free of charge, and held at times chosen by the GP surgeries. This complies with Norwegian regulations for mandatory seminars for GPs to become or to remain a specialist. The first session is 3 hrs, the rest 4 hrs. The first three sessions are delivered during 4-6 weeks, the final session after an interval of about 8 weeks.

	schedule, and their duration, intensity or dose.	
**9.**	**TAILORING**<br><br>If the intervention was planned to be personalised, titrated or adapted, then describe what, why, when, and how.	• The seminar plan has been adjusted according to feedback from GPs in the planning process, and is designed to fit their needs both professionally and practically (content, pedagogical strategies, timing and location). The organizers will ask for feedback both for ongoing tailoring and for research needs.

Supplementary Table 3. Full programme of the clinical seminar series on alcohol related health problems

ALCOHOL-RELATED HEALTH PROBLEMS IN GENERAL PRACTICE

CLINICAL TOPIC SEMINAR AT THE GROUP PRACTICE – FOUR SEMINARS WITH HOME ASSIGNMENTS

ADD LOCATION AND DATES

SEMINAR 1 (3X45 MIN), DOCTORS AND EMPLOYEES

ADD FACILITATOR NAMES

- WELCOME AND INFORMATION ABOUT THE SEMINAR.
- INTRODUCTION TO *ENDRE*, WEB-BASED ADJUSTMENT SUPPORT FOR PATIENTS. DISCUSSION OF USE OF ENDRE IN OWN PRACTICE.
- RELATIONSHIP BETWEEN ALCOHOL CONSUMPTION, HEALTH AND ILLNESS IN GENERAL PRACTICE. PRAGMATIC CASE FINDING. CONDITIONS WHERE ALCOHOL HAS AN IMPACT. ADDICTIVE PRESCRIPTION DRUGS. BARRIERS WHEN TAKING ABOUT ALCOHOL. WHAT DOES PATIENTS THINK OF BEING ASKED ABOUT ALCOHOL?
- INTRODUCTION INTO AGEING AND ALCOHOL CONSUMPTION.
- HOME ASSIGNMENT – CONDITIONS WHERE ALCOHOL HAS AN IMPACT.
- SUMMARY AND COMMENTS.

SEMINAR 2 (4X45 MIN) – DOCTORS AND EMPLOYEES

ADD FACILITATOR NAMES

- *ENDRE* – EXPERIENCES AND FRUSTRATIONS.
- ALCOHOL – EASIER THAN YOU THINK (3X45 MIN). SHORT ON EXPERIENCES WITH THE HOME ASSIGNMENT. DIFFERENT HEALTH EFFECTS OF ALCOHOL. PRACTICAL STRATEGIES FOR THE GP. CLINICAL EXAMPLES AND EVIDENCE. GP`S TOOLBOX. WHITE PERIODS, “HALVING TEST”. AGEING AND ALCOHOL.
- BRIEF GROUP DISCUSSION ON HOW TO START A DIALOGUE ON ALCOHOL WITH YOUR PATIENT.

- HOME ASSIGNMENT FOR NEXT TIME – STRATEGIES TO PREPARE AND START CONVERSATIONS ON ALCOHOL.
- SUMMARY AND COMMENTS.

SEMINAR 3 (4X45 MIN) – DOCTORS AND EMPLOYEES

ADD FACILITATOR NAMES

- *ENDRE* - EXPERIENCES AND FRUSTRATIONS.
- ALCOHOL – EASIER THAN YOU THINK (2X45 MIN). SHORT ON EXPERIENCES WITH THE HOME ASSIGNMENT. FOLLOW-UP ON PREVIOUS SEMINAR.
- MOTIVATION AND CHANGE (60MIN) WITH PRACTICAL EXERCISES.
- HOME ASSIGNMENT FOR NEXT TIME – TOOLS FROM YOUR TOOLBOX.
- SUMMARY AND COMMENTS.

SEMINAR 4 (4X45 MIN) – DOCTORS AND EMPLOYEES

ADD FACILITATOR NAMES

- *ENDRE* – EXPERIENCE AND FURTHER USE – IS IT A USEFUL TOOL?
- BRIEF DISCUSSION ON HOME ASSIGNMENT, REFLECTIONS AND SHARING OF EXPERIENCES.
- MOTIVATION AND CHANGE (45MIN) – PART 2.
- ETHICAL AND PRACTICAL PROBLEMS RELATED TO DRIVER`S LICENSE (45MIN).
- SHORT ON ADDICTIVE PRESCRIPTION DRUGS AND ALCOHOL (30MIN).
- COLLABORATION AND REFERRAL TO OTHER ACTORS IN THE MUNICIPAL AND SPECIALIZED HEALTH SERVICES (30 MIN).
- EVALUATION.

SEMINAR LEADER: ADD NAME

LECTURERS/MODERATORS:

- ADD NAMES
